# Evaluation of altered starch mutants and identification of candidate genes responsible for starch variation in wheat

**DOI:** 10.1186/s12870-023-04389-3

**Published:** 2023-08-01

**Authors:** Ahsan Irshad, Huijun Guo, Hongchun Xiong, Yongdun Xie, Hua Jin, Jiayu Gu, Chaojie Wang, Liqun Yu, Xianghui Wen, Shirong Zhao, Luxiang Liu

**Affiliations:** 1 Institute of Crop Sciences, National Engineering Laboratory of Crop Molecular Breeding, Chinese Academy of Agricultural Sciences, National Centre of Space Mutagenesis for Crop Improvement, Beijing, 100081 China; 2 Key Laboratory of Molecular Medicine and Biotherapy, School of Life Sciences, Beijing Institute of Technology, Beijing, 100081 China

**Keywords:** Functional genes, Mutation, Quality traits, Starch, Wheat

## Abstract

**Background:**

Induction of mutation through chemical mutagenesis is a novel approach for preparing diverse germplasm. Introduction of functional alleles in the starch biosynthetic genes help in the improvement of the quality and yield of cereals.

**Results:**

In the present study, a set of 350 stable mutant lines were used to evaluate dynamic variation of the total starch contents. A megazyme kits were used for measuring the total starch content, resistant starch, amylose, and amylopectin content. Analysis of variance showed significant variation (p < 0.05) in starch content within the population. Furthermore, two high starch mutants (JE0173 and JE0218) and two low starch mutants (JE0089 and JE0418) were selected for studying different traits. A multiple comparison test showed that significant variation in all physiological and morphological traits, with respect to the parent variety (J411) in 2019–2020 and 2020–2021. The quantitative expression of starch metabolic genes revealed that eleven genes of JE0173 and twelve genes of JE0218 had consistent expression in high starch mutant lines. Similarly, in low starch mutant lines, eleven genes of JE0089 and thirteen genes of JE0418 had consistent expression in all stages of seed development. An additional two candidate genes showed over-expression (*PHO1, PUL*) in the high starch mutant lines, indicating that other starch metabolic genes may also contribute to the starch biosynthesis. The overexpression of *SSII, SSIII* and *SBEI* in JE0173 may be due to presence of missense mutations in these genes and *SSI* also showed overexpression which may be due to 3-primer_UTR variant. These mutations can affect the other starch related genes and help to increase the starch content in this mutant line (JE0173).

**Conclusions:**

This study screened a large scale of mutant population and identified mutants, could provide useful genetic resources for the study of starch biosynthesis and genetic improvement of wheat in the future. Further study will help to understand new genes which are responsible for the fluctuation of total starch.

**Supplementary Information:**

The online version contains supplementary material available at 10.1186/s12870-023-04389-3.

## Introduction

The quality of wheat flour evaluates based on endosperm starch contents and its composition. The endosperm starch that is approximately 70% dry weight of seed has significant effect on the yield and quality with showing capacity of sink tissues in wheat [1]. The starch granule is developed by composition of amylose (25%) and amylopectin (75%) [2]. By using different breeding approaches or through mutations in genes, we can change the percentage of amylose and amylopectin in resources and improve the quality of wheat. Such as suppression of *GBSSI* gene to produce partial waxy wheat to create less amylose content which help in production of good quality noodles [3]. By using different approaches such EMS treatment, gene editing and functional genomics tools provide high starch wheat varieties [4, 5]. Similarly, with the increment of amylose content in wheat help to provide additional health benefit such as obesity, cardio-vascular and brain tissues disease [6].

Glucose-1-phosphate play important role in the amyloplast during starch biosynthesis. The initiation of starch biosynthesis by ADP-glucose pyrophosphorylases (AGPase) with help of other enzymes (SSs, SBEs, GBSS, ISA) in the seed [6]. The formation of amylose initiated by granule bound starch synthase (GBSS) and amylopectin formation done with the coordination of starch synthase, starch branching enzymes and debranching enzymes [7]. Different genes involved in the modulation of amylose and amylopectin ratio during starch biosynthesis by using different breeding and biotechnological approaches in which gene editing, transposon, insertion, and RNAi [8]. There was decline in total starch and amylopectin content due to missense mutation in the *TaAGPL1-A*, while overexpression of *TaGPL1* and *TaGPS1* helped to increase total starch and thousand grain weight in wheat [9]. A waxy wheat produced due to knockout of all three genes of *GBSSs* and all the starch mainly composed of amylopectin in the endosperm of wheat [10]. Through RNAi for *SSI* in wheat help to increase the concentration amylose content. Similarly, inactivation of *SSII* also increased the amylose content in wheat [11] The mutation in *TaSSIVb-D* had no significant affect at the reserve starch but there was less granules in leaves [7]. Similarly, due to double mutant (*agp.L-B1/ssIVb-D*), grain starch and amylopectin contents were less than other single mutants in wheat [7]. The formation of starch is a complex process and identified candidate genes with unknown factors help in the formation of reserve starch in the cereal crops [12].

Phenotypic variation has been produced in cereal crops through chemical agent. One of the most frequently use chemical agent is ethyl methane sulphonate (EMS) in cereal crops [5]. EMS works as an alkylating agent by affecting Guanine (G), due to which mispairing with thiamine (T) instead of cytosine (C) and transition happen from G/C to A/T. EMS mutation is preferable than other biotechnological approaches because it provides multiple alleles of a specific gene within the small population. Many starch biosynthesis genes have been used to produce novel variations through EMS-induced mutagenesis. Through EMS mutation, partial and complete waxy wheat had been produced by targeting the *GBSSI* gene [13]. Furthermore, mutation in *SSIIa, SBEIIa* and *SBEIIb* used to produce high and low starch mutants [9]. To find out new genes/QTL is a challenging task from the natural population [14]. So for this there is need to make near isogenic lines or other functional genomic tools such as genome editing [15].

**Fig. 1:**
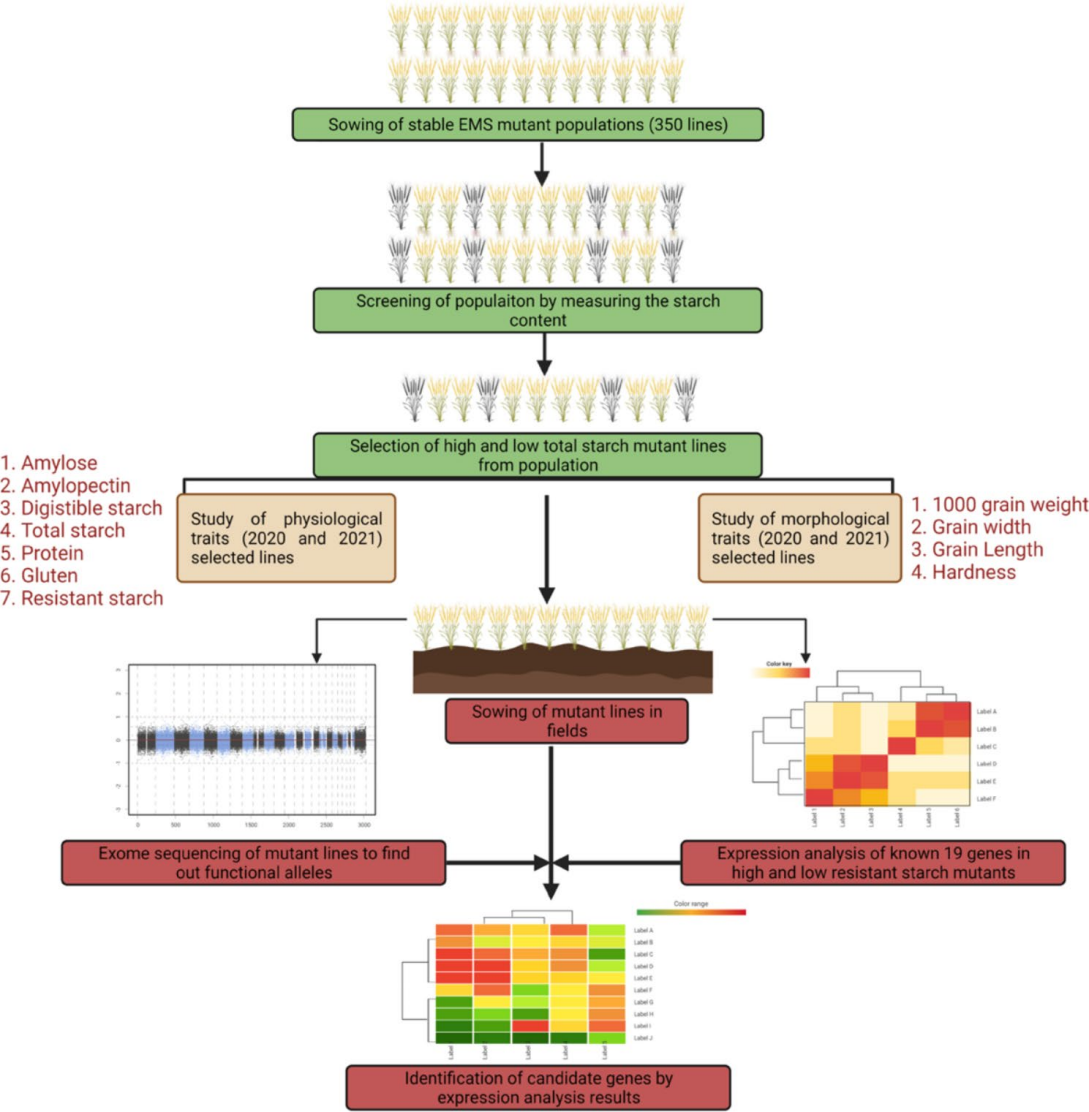
Overall workflow figure. The green part is showing the screening of mutant; light brown is showing the traits studied by two years screening; and dark red part is showing the expression and exome sequencing of the selected mutants

In the present study, a set of 350 stable mutant lines were screened for total starch content. The parent variety of this stable population is J411. Further, two high starch mutant lines and two low starch mutant lines were used to study quantitative gene expression patterns of 19 starch metabolic pathway genes during seed development. The exome sequence had been done to know the allelic variations in these selected lines. Overall workflow is given in the Fig. 1.

## Results

### Screening of mutants with altered seed starch content

**Fig. 2:**
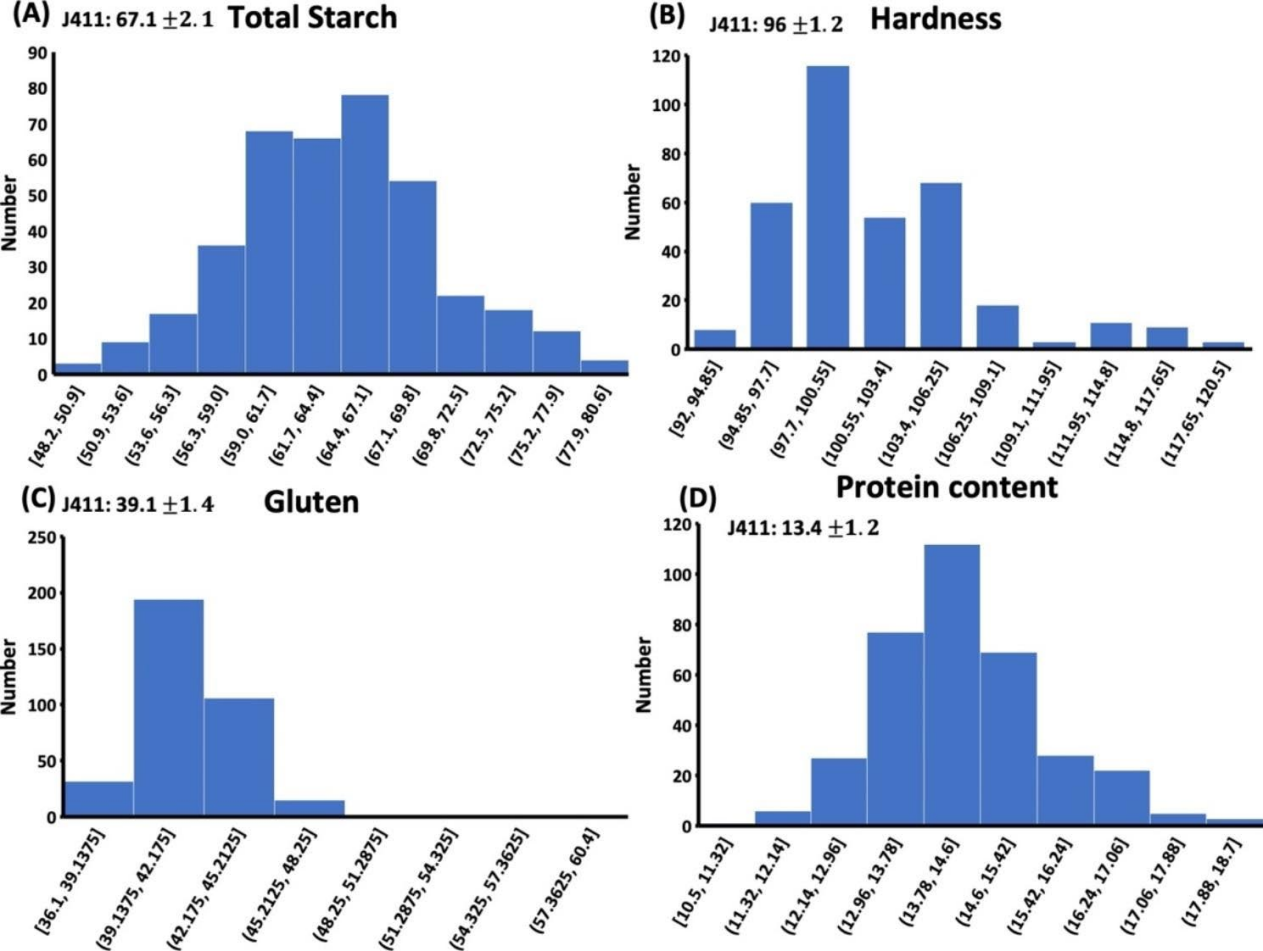
Physiological and morphological variations in 350 stable mutant lines. Starch content variations in the stable population. Bold type indicates the variation in the WT (J411) (**A**), Hardness in the mutant seeds (**B**), Gluten profile in the mutant population (**C**) and Protein content in the mutant seeds. Bold type indicates the average of protein content in wild-type plants (**D**)

Through starch analysis, it’s indicated that the total starch contents in mutant lines were stable and consistent. Similarly, analysis of variance showed significant variation between the mutant lines. Most of lines showed significant variations (60%) for total starch contents from the parent variety. The lowest value for total starch content was 48.2% and the highest value in the mutant lines was 80.6%, while the value of WT (J411) was 67.1%. Gluten, Protein, and hardness of seed also showed variation within the population. The lowest seed hardness value was 92% and highest was 120% while the value of WT was 96%. The maximum mutant lines whose values were 39% for gluten while the minimum value for protein was 14%. The results of these 350 lines are given in Fig. 2.

### Identification of high and low starch lines

Two high starch (JE0173 and JE0218) and two low starch (JE0089 and JE0418) mutant lines had been selected from mutant population. The average low starch was 50% and 52% in two mutant lines JE0089 and JE0418 respectively for both year data while highest starch was 76% and 77% in mutant lines JE0173 and JE0218 respectively, and average value of J411 was 66% in 2020 and 2021. These lines showed significant difference from J411 in two years data. Further, amylose content of JE0089 was significantly lower in both year of data while amylose content of high starch mutant line JE0218 was higher as compared to WT but there was lower amylose content for JE0173 mutant line. Similarly, amylopectin content of all mutant lines showed significant difference from WT. The resistant starch of high starch mutant lines showed significant difference from WT and both lines had higher resistant starch contents than low starch mutant lines. Highest resistant starch was observed in the JE0218 line. There was significant difference in all mutant lines as compared to WT for digestible starch. Protein contents was also higher in the high starch mutant lines. Similarly, gluten was high in high starch lines and less in less starch lines. Clear and significant differences (*p* < 0.05) were recorded in the case of physiological and biochemical parameters. The results of two years selected lines have been given in the Table 1.

### Phenotypic characteristics of mutants

**Fig. 3:**
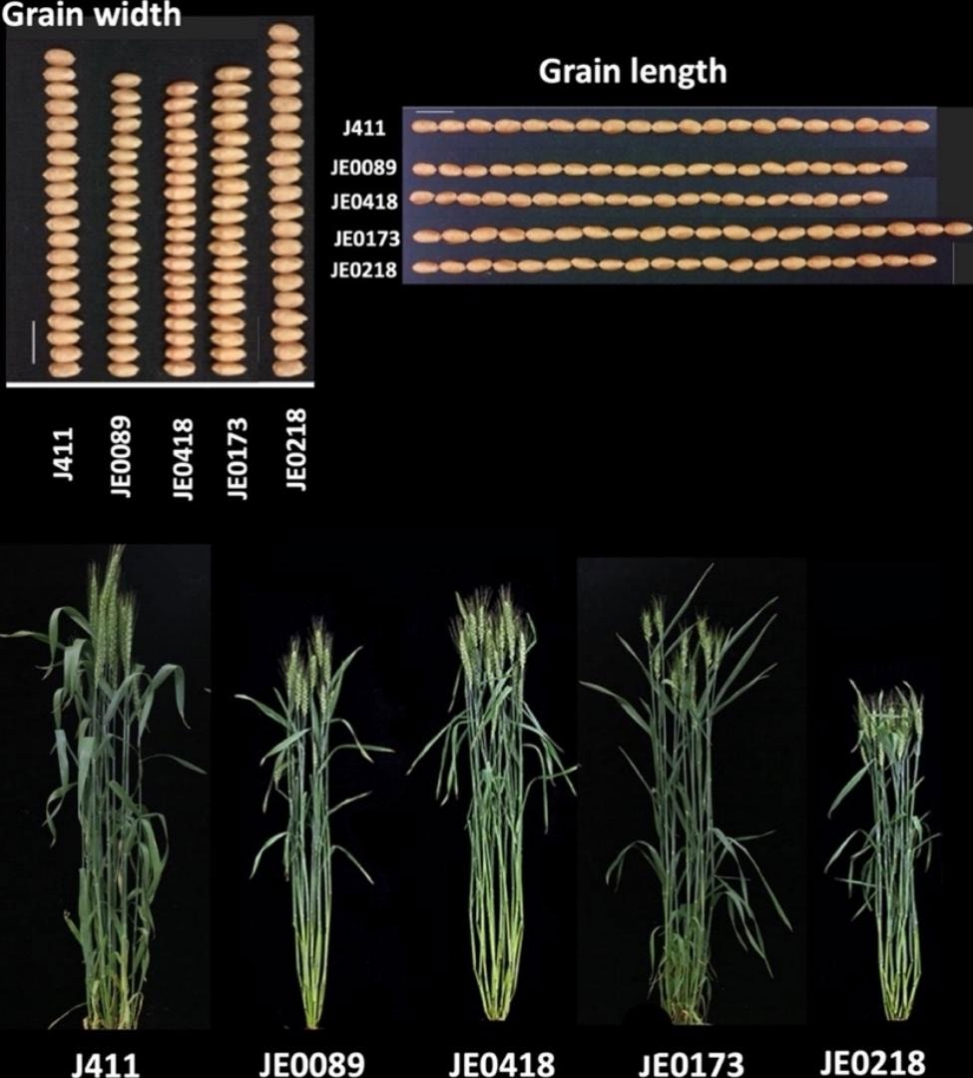
Phenotypic variation on grains and plants observed at maturity stage. The image of grain width and grain length in the high starch (JE0173 andJE0218) and low starch mutants (JE0089 and JE0418) as compared to WT (J411). Plants of high and low starch mutants

By taking pictures of the WT(J411) and mutant plants of wheat at heading stage, the mature plants were investigated for different biological traits, and the mature grains were photographed (Fig. 3). Grain length and grain width showed in the images of low starch mutants (JE0089 and JE0418) and high starch mutants (JE0173 and JE0218) with comparison of WT (J411). Clear and significant differences (*p* < 0.05) were recorded in the case of morphological parameters. The results showed that low starch mutants and high starch mutants had significant effects on the four yield-related traits in wheat, namely, TGW, grain width, grain length and grain area in two years data. The grain width of JE0218 was higher than all mutant lines, while the Grain length of JE0173 was higher than all mutants. The TGW was higher of both high starch mutants in which the average TGW of JE0173 was 45.4 g and JE0218 had 46.6 g, while WT had 40.2 g. The gluten content of JE0218 was also higher than all other lines in both year of data (Table 2; Fig. 3).

**Table 1:** Different physiological traits study in high starch (JE0173 and JE0218) and low starch mutants (JE0089 and JE0418) in 2020 and 2021 years

		Wild type	High starch mutants		Low starch mutant	
Years	Traits	J411	JE0173	JE0218	JE0089	JE0418
**2019–2020**	TS (% WF)	67.1±0.7	77.8±0.4**	76.12±0.3**	51.69±0.8**	54.45±0.6**
	PC (%)	13.4±0.3	15.4±0.5*	15.9±0.2**	12.2±0.3	10.5±0.7*
	AC (% WF)	27.4±0.1	28.78±0.2	30.12±0.5**	26.34±0.6*	27.83±0.4
	APC (% WF)	72.6±0.9	71.22±0.4	69.88±1.2*	72.12±0.7	72.17±0.6
	RS (% WF)	0.95±0.1	1.87±0.0**	3.68±0.1**	1.24±0.1*	1.11±0.2
	DS (% WF)	65.05±0.9	76.93±0.9**	72.44±1.1**	50.45±1.1**	52.34±0.6**
	Gluten (%)	39.9±0.9	41.2±1.2*	43.5±0.6**	38.7±1.4*	36.1±0.4**
**2020–2021**	TS (% WF)	68.2±0.3	75.3±0.6**	77.12±0.8**	53.22±1.2**	52.4±0.6**
	PC (%)	13.9±0.4	15.7±0.3**	15.1±0.8**	12±0.4*	12.1±0.1*
	AC (% WF)	26.8±0.3	27.78±0.4	29.11±0.2**	25.44±0.9*	28.81±0.7**
	APC (% WF)	73.2±0.9	72.22±1.4	70.89±0.3**	74.56±0.5*	71.10±0.8*
	RS (% WF)	0.89±0.0	2.11±0.3**	4.12±0.2**	1.38±0.1*	1.51±0.3*
	DS (% WF)	64.44±0.2	74.5±0.6**	73±1.0**	51.84±1.7**	49.89±0.4**
	Gluten (%)	39.6±0.6	40.85±0.9*	44.2±1.4**	39.1±0.5	36.8±0.9**

Values reported are means SD. The phenotype data of each genotype were compared to that of WT using the two-tailed Student’s t-test. Asterisks indicate probability values in Dunnett tests of mutant lines against the wild type (*P < 0.05 and **P < 0.01). TS: Total starch; % WF: Percentage of whole flour; PC: Protein content; AC: Amylose content; APC: Amylopectin Content; RS: Resistant starch; DS: Digestible starch

**Table 2:** Incidence of phenotypic variants observed from high and low starch mutants

		Wild type	High starch mutants		Low starch mutant	
Years	Traits	J411	JE0173	JE0218	JE0089	JE0418
**2019–2020**	TGW (gram)	40.140±0.4	43.81±0.2**	45.39±0.6**	39.12±0.2*	37.5±0.4**
	Grain Area (mm)	15.46±0.5	18.04±0.7*	17.76±0.2*	14.47±0.9	12.33±0.4*
	Grain Width (mm)	4.07±0.1	4.1±0.3	4.44±0.1*	4.02±0.2	3.98±0.3**
	Grain length (mm)	3.8±0.2	4.4±0.1**	4±0.1*	3.6±0.3**	3.1±0.1**
	Grain Hardness (%)	97±0.9	98±1.2	99±0.6*	93±0.6**	92±0.9**
**2020–2021**	TGW (gram)	41.12±0.7	44.92±0.6**	46.12±1.2**	40.11±0.4*	39.12±0.3*
	Grain Area (mm^2^)	15.2±0.4	18.9±0.2*	18.45±0.7*	13.93±0.4*	12.43±0.9*
	Grain Width (mm)	4.11±0.2	4.2±0.1*	4.5±0.3*	3.98±0.1*	4.01±0.1
	Grain length (mm)	3.7±0.1	4.5±0.1**	4.1±0.2*	3.5±0.1*	3.1±0.3*
	Grain Hardness (%)	98±0.6	99±0.9*	96±0.7*	94±0.3*	93±1.4**

Values reported are means SD. The phenotype data of each genotype were compared to that of WT using the two-tailed Student’s t-test. Asterisks indicate probability values in Dunnett tests of mutant lines against the wild type (*P < 0.05 and **P < 0.01). TGW: Thousand grain weight; mm: millimeter

### Impact at morphology of starch granules in selected mutants

**Fig. 4:**
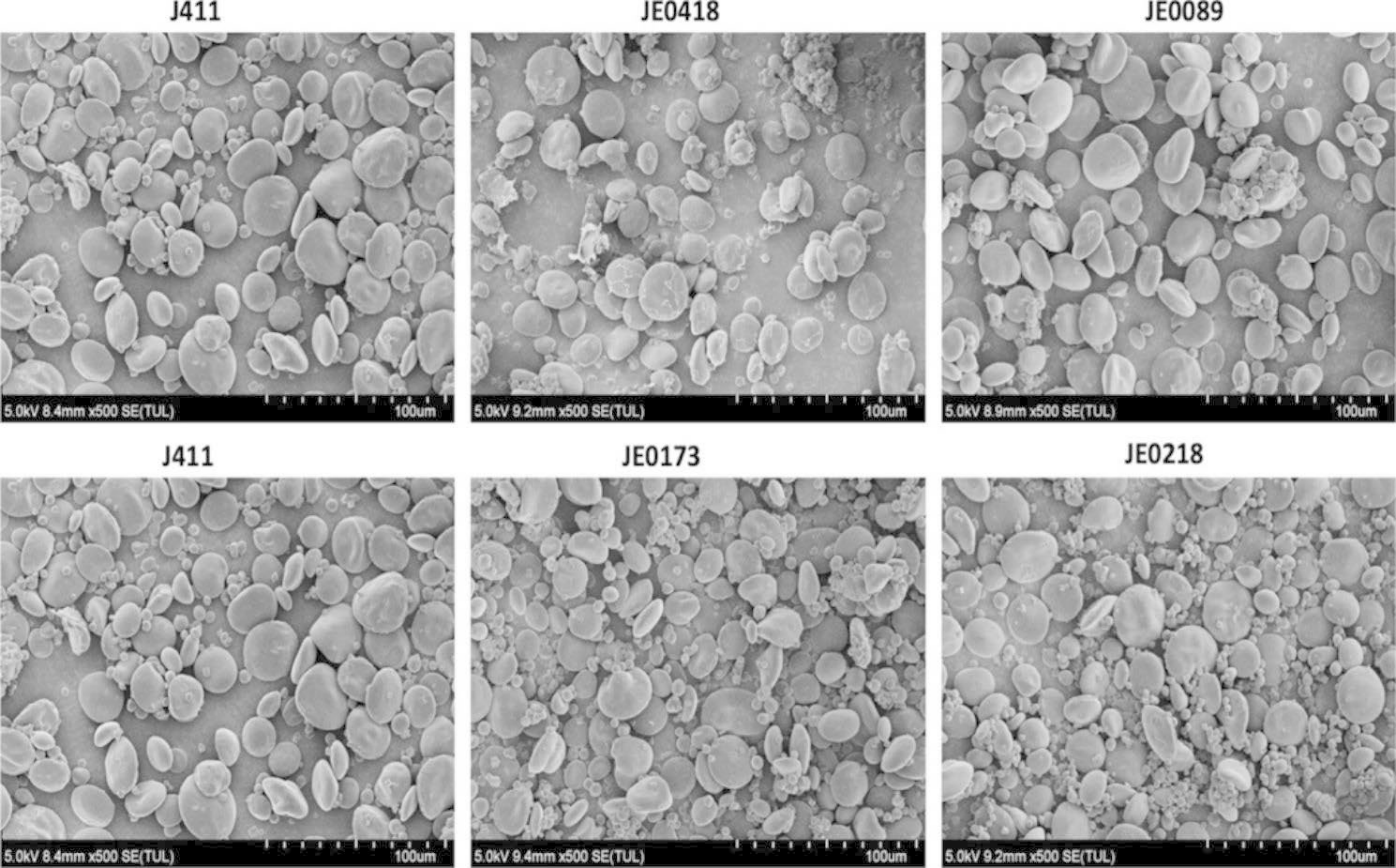
Starch granule structure of wheat grains. Starch structure from parental line J411, low starch mutants; JE0418, JE0089 and high starch mutants; JE0173, JE0218 visualized by SEM. For each mutant line, at least two grains were examined, and similar results were observed

TM4000 scanning electron microscopy was used to find out the difference of grain morphology between WT (J411) and high starch mutants (JE0173, JE0218) and low starch mutants (JE0089, JE0418) (Fig. 4). The pure starch was extracted from the seeds by crushing them in the grinder. It was observed that in the low starch mutants (JE0089 and JE0418) that the starch granules were reduced and decreased in numbers with less A-granules and B-granules (Fig. 4). Similarly, there was significant difference in size and number of grains as compared to WT in the high starch mutant lines (JE0173 and JE0218). The number of A- granules and B-granules were higher in the high starch mutant lines.

### Expression pattern of starch metabolic genes in high starch mutant lines

**Fig. 5:**
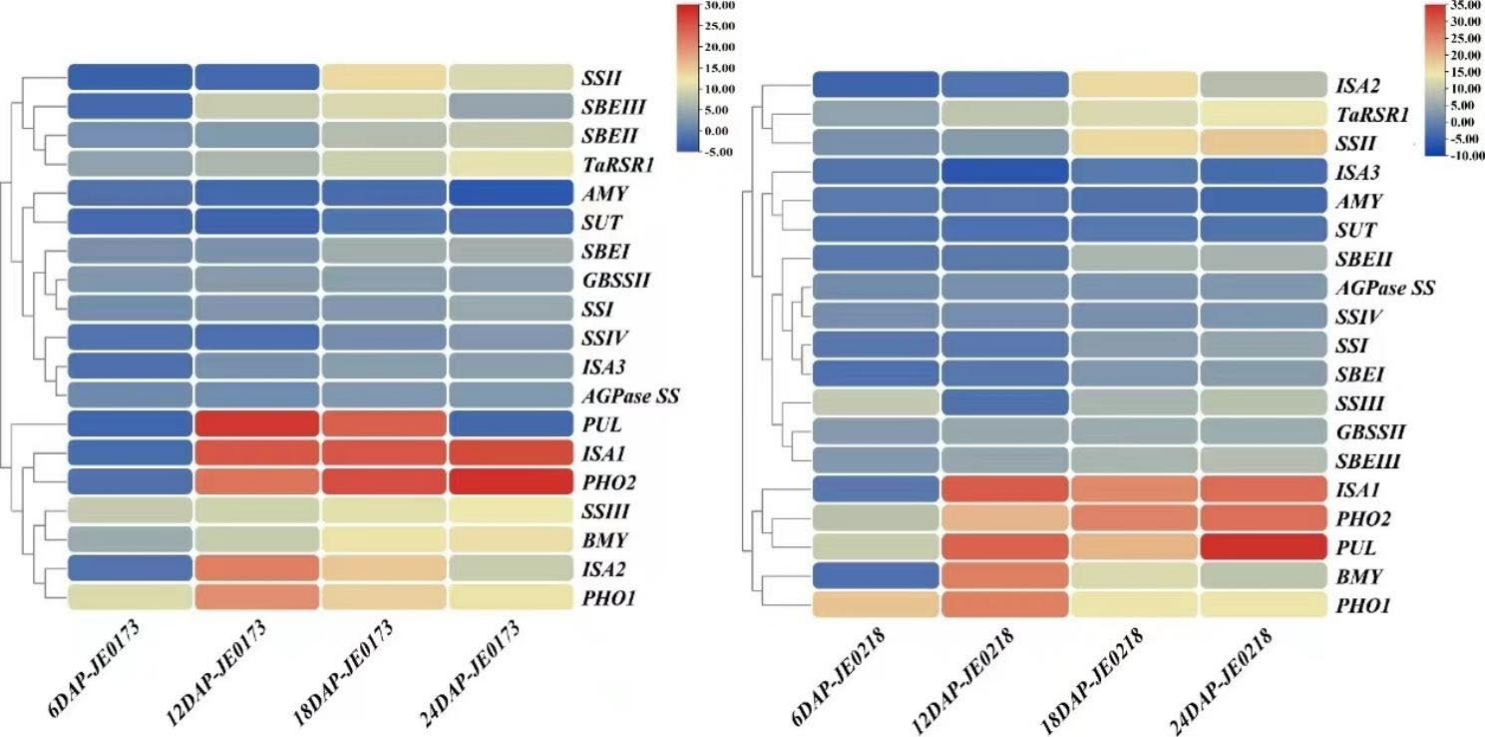
Expression levels of starch metabolic genes in high starch mutants. Expression levels of wheat starch genes in two high starch mutant lines (JE0173 and JE0218) at 6, 12, 18 and 24 days after pollination (DAP) in wheat developing grains. The expression levels for each line was calibrated as expression folds

The comparatives expression of nineteen genes in high starch mutant lines (JE0173 and JE0218) showed that eleven genes of JE0173 and twelve genes of JE0218 had consistent expression in all stages of seed development (Fig. 5). While eight genes of JE0173 and seven genes of JE0218 expression pattern was inconsistent in four stages of seed development. From these eleven genes of JE0173, eight genes showed overexpression at four seed development stages and three genes (*AMY, BMY, SUT*) showed reduced expression in all stages while, from 12 genes of JE0218, nine genes showed overexpression and three genes (*AMY, ISA3, SUT*) had reduced expression (Fig. 5).

### Expression pattern of starch metabolic genes in low starch mutant lines

**Fig. 6:**
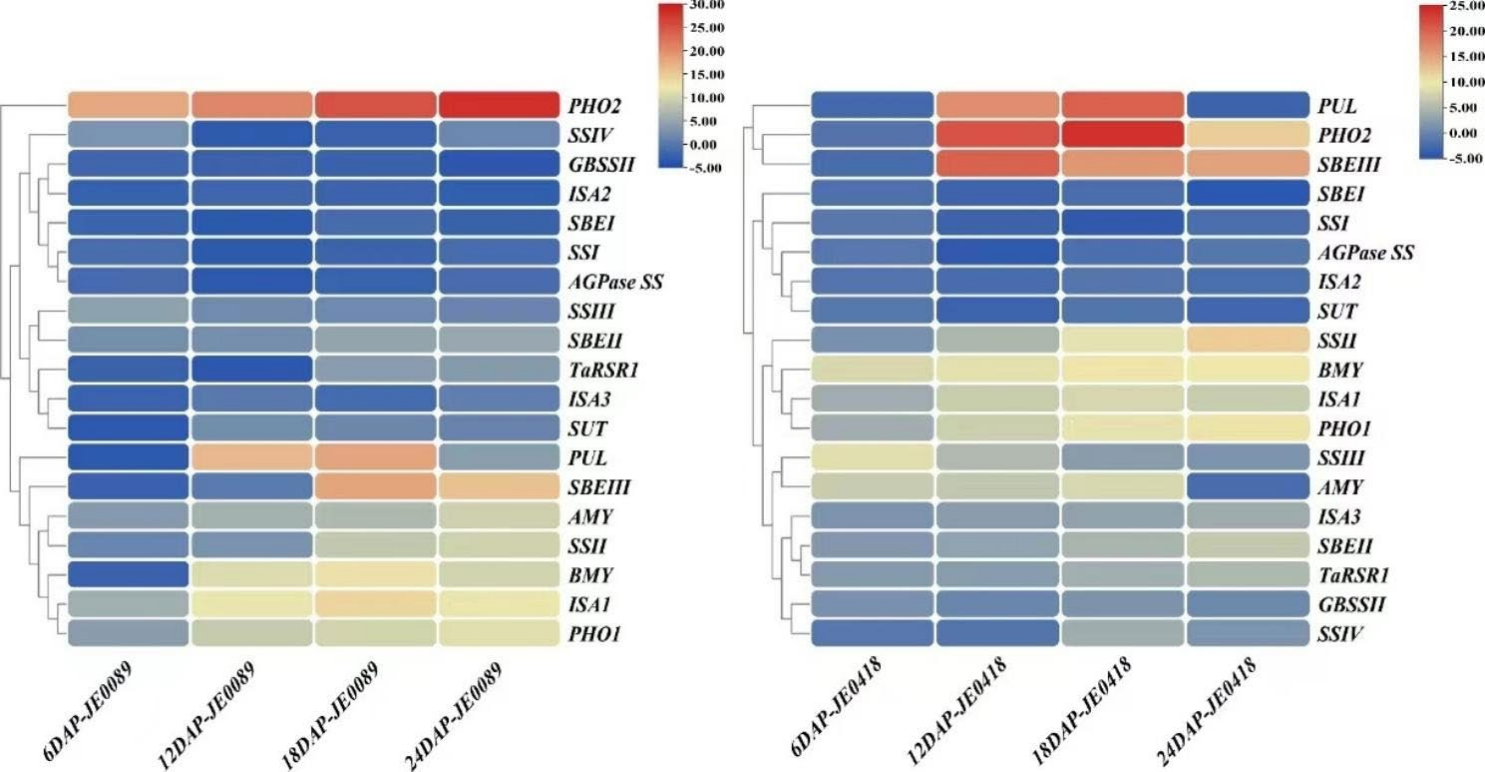
Expression of starch metabolic genes in low starch mutants Expression levels of wheat starch genes in two low starch mutant lines (JE0089 and JE0418) at 6, 12, 18 and 24 days after pollination (DAP) in wheat developing grains. The expression levels for each line was calibrated as expression folds

The low starch mutant’s expression analysis had been conducted. The quantitative gene expression analysis showed that from nineteen genes of starch biosynthesis, eleven genes of JE0089 and thirteen genes of JE0418 had consistent expression in four stages of seed development (Fig. 6). Five genes (*GBSSI*, *SSI, SBEI, ISA2, AGPase S*) of JE0089 showed reduced expression and six genes (*SSIII, SBEII, AMY, ISAI, PHO1, PHO2)* had over expression from these eleven genes. Similarly, from the thirteen genes of JE0418, six genes (*SSI, SBEI, ISA2, SUT AGPaseS, SSIV)* had reduced expression while seven genes (GBSSII, *SSIII, SBEII, BMY, ISA1, PHO1, SSII*) had over-expression in all seed development stages. The expression pattern of remaining starch synthesis genes in both mutant lines were inconsistent by showing high and reduced expression in different stages of seed development. In the present study, the low expression of *TaRSR1* in the low starch mutant lines may be this gene had no effect in starch metabolism. This differential expression analysis in four mutants give the idea that there is involvement of other starch metabolic pathway genes such as phosphorylase, isoamylase etc., in the starch biosynthesis.

### Comparison of SNPs and indels of the wheat mutant lines

Exon sequencing had been done for selected high starch mutant lines (JE0173 and JE0218) and low starch mutant lines (JE0089 and JE0418). It was depicted that in the mutant lines, the number of SNPs were more in all four mutant lines than Indels. The number of SNPs in JE0418 was higher than other three mutants. But Indels were almost in equal amount in all mutant lines. The different types of SNPs between the mutants suggested that there were more transitions than transversions in the four mutants (figure S1). There were also fewer synonymous mutations in all mutant lines (figure S1).

From four mutant lines, twenty-two variants were identified in nineteen starch biosynthesis genes. Ten variants were discovered in JE0173 in which four were missense mutation (*SSIII, SBEI, GBSSI, SSII*) and four were synonymous mutation (*ISA1, PHO2, TaRSR1, SBEIII*) while remaining two were 3_prime_UTR variant (*SSI*) and splice region variant (*SSII*). JE0218 had two variants that consist of one synonymous (*AMY*) and other was missense (*TaRSR1*) mutations. Similarly, JE0089 and JE0418 had six and four variants respectively. (Table 3). Three missense (*SSI, PHO2, PUL*) mutations in JE0089 and two (*SBEIII, SSII*) in the JE0418 had been observed, while two were synonymous in both lines.

The overexpression of *SSII, SSIII* and *SBEI* in JE0173 may be due to presence of missense mutations in these genes and *SSI* also showed overexpression which may be due to 3-primer_UTR variant. These mutations can affect the other starch related genes and help to increase the starch content in this mutant line (JE0173). Similarly, in JE0218, there was missense mutation in *TaRSRI* and its showed overexpression as compared to WT (J411). In the low starch mutant line JE0089, *SSI* and *PUL* had reduced expression and these genes had missense mutation. *PUL* also had missense mutation, but the expression of this gene was inconsistent. So, these genes which showed reduced expression and other starch related genes might be the responsible for low starch contents in this mutant line. Similarly, in JE0418, *SBEIII* had reduced expression due to missense mutation and this mutation might be responsible for low starch content in this mutant line.

**Table 3:** Allelic variation in the starch metabolic genes in two high and two low starch mutants

Mutant lines	Gene name	Gene ID	Mutation type	position	Nucleotide change	Amino acid change	Chromosome
**JE0173**	*GBSSI*	TraesCS2B03G0997500	Missense	1962	G/A	Val/ile	2B
	*SSI*	TraesCS7D03G0264000	3_prime_UTR_variant	9993	G/A	Lue/Lys	7D
	*SSIII*	TraesCS2B03G1238800	Missense	6421	C/T	Thr/His	2B
	*SBEI*	TraesCS7D03G1261200	Missense	2429	C/A	Pro/His	7D
	*ISA1*	TraesCS7D03G0559000	Synonymous	620	G/A	No change	7D
	*Pho2*	TraesCS3A03G087060	Synonymous	5754	G/A	Val/Leu	3 A
	*TaRSR1*	TraesCS1B03G0192900	Synonymous	3270	G/A	No cahnge	1B
	*SBEIII*	TraesCS7A03G0826800	Synonymous	16,500	C/T	Leu/Phe	7 A
	*SSII*	TraesCS1A03G0361500	Missense	3324	G/A	Ser/Asn	1 A
	*SSII*	TraesCS5D03G1065400	splice_region_variant	2452	G/A	Stop codon	5D
**JE0218**	*AMY*	TraesCS5A03G1096000	Synonymous	1337	C/T	No cahnge	5 A
	*TaRSR1*	TraesCS1B03G0192900	Missense	947	G/A	Val/Leu	1B
**JE0089**	*SSI*	TraesCS6B03G0953800	Missense	891	C/T	Ser/Pro	6B
	*BMY*	TraesCS2D03G0467600	3_prime_UTR_variant	288	C/T	No change	2D
	*Pho1*	TraesCS5D03G0900800	Synonymous	3555	C/T	No change	5D
	*Pho2*	TraesCS3A03G0870600	Missense	5734	C/T	Pro/Leu	3 A
	*PUL*	TraesCS7D03G029750	Missense	13,940	C/T	Pro/Ala	7D
	*SSII*	TraesCS6D03G0680800	Synonymous	5573	G/A	Leu/His	6D
**JE0418**	*AMY*	TraesCS5A03G1096000	Synonymous	980	G/A	No change	5 A
	*SBEIII*	TraesCS7A03G0826800	Missense	49	G/A	Stop codon	7 A
	*SSII*	TraesCS1A03G0361500	Missense	11,434	G/A	Ser/Asn	1 A
	*SSII*	TraesCS7D03G0426300	Synonymous	955	C/T	Pro/Leu	7D

## Discussion

Modification of starch composition in wheat help to increase starch content and yield of the crop which ultimately effect on the public health due to good quality of flour. Different chemical mutagenesis have been used in different crop for studying the functional genomic and breeding [16]. Different research has proved that using mutants with altered phenotypes help to know the function of specific gene in an organism. In the present study, 350 stable mutants were used for screening total starch contents and find out low starch mutant lines and high starch mutant lines from this stable mutant population. Different literature proved that EMS treated lines were used to find out mutations for candidate genes in diploid, tetraploid, and hexaploid wheat [17–19]. Alteration of starch contents in different cereal crops especially in wheat is getting more attention because starch play important role in the food and non-food applications. By using EMS mutant stable lines which contained multiples variations in gene pool can help us to find out the functional mutations in the candidate genes which can be difficult to find in natural population due to limited variations in natural population [17]. These mutagenesis can directly applied to superior elite cultivars and backcrosses with parent variety help to remove unnecessary mutation by introducing novel allele in to the new line [20].

The deposition of protein increased or decreased due to mutation and there will be significant reduction of amino acids in seed tissues exposed to EMS chemical [21]. The deposition of protein in grain filling stage of wheat is earlier than starch deposition in grain filling stage which normally start after 12 days of anthesis, [22]. The deposition of protein in cereal crops normally initiated 8 to 10 days after anthesis and reaches its peak position in 20 days while deposition of starch start at the same time but its complete deposition need at least 45 days [23]. Hence it is depicted that starch and protein deposition in the cereal is an independent event which controlled by sink and source limited process respectively. Recent studies have proved that not only plant hormone transduction but also starch and sucrose metabolism play significant role in the grain size and grain weight during grain development in the cereal [24]. In the present study, the grain size and 1000 grain weight of high starch lines were higher than the WT. Normally, the total starch contents and TGW normally increase in initial grain filling stage but totally ceased at the end of grain filling stage. Similarly in the present study the correlation between total starch and TGW and between total starch and resistant starch showed similar results as reported in the previous studies [13].

The key genes of starch biosynthesis such as *AGP-S1*, *SS1*, *SSIIa*, *SSIIIa*, *SBEIIa*, *SBEIIb*, and *GBSSIa* expression level play significant role in starch biosynthesis and accumulation. Changing of expression pattern of these main genes may have positive affect on the grain weight of the wheat during grain filling stages [25, 26]. In the present result, high starch mutants have higher expression of the main genes (*GBSSII, SBEII, AGPaseSS* and *SSs)* as compared to low starch mutants during grain filling stages may lead to accumulation of high starch [27]. Amylose content increase due to overexpression of *GBSSI*. Similarly, due to silent and null mutants produce waxy and partial waxy wheat with low amount of total starch and lacking amylose contents [28, 29]. With the inhibition of *ISA* help to reduction in the amylopectin contents in wheat [30], while silencing of *SBEII* also increased amylose content with reduced total starch contents [31]. Therefore, these results support that the overexpression of main starch genes help to increase total starch contents in high starch mutant lines.

Amylases (*AMY* and *BMY*) with phosphorylases (*Pho1* and *Pho2*) play significant role in starch metabolism along with hydrolysis and degradation [32]. These are the main genes which play important role in the starch structure and their grain morphology. Alteration of starch structure was observed in rice and potato by silencing of these genes [33]. Similarly, overexpression of *AMY* and *BMY* fluctuate the starch structure and baking quality in wheat [34]. A negative transcription factor (*RSR1*) had been observed during co-expression in the rice which negatively regulate the some starch biosynthesis genes and modulate the starch metabolism [35]. Therefore, it can say that the accumulation of amylopectin in low starch mutant may be the overexpression of key genes of starch biosynthesis. The differential expression of starch main genes in low starch mutants and high starch mutants give the idea that the involvement of other starch metabolic genes such as phosphorylase during synthesis of starch.

## Material and method

### Plant material

In 2019–2020, a stable mutant population that consist of 350 lines, were grown in the field with three replications at the Institute of Crop Sciences, Chinese Academy of Agricultural Sciences, Beijing. This mutant population was developed by Institute of Crop Sciences, Chinese Academy of agricultural Sciences by using the ethyl methane sulphonate chemical (EMS) and other mutagens to the J411 variety. J411 is the hexaploidy modern cultivar in China. After selecting the lines based on different traits, stable lines were developed. After maturing, seed samples from each mutant lines were collected and crushed these seeds to make the flour for studying the total starch contents and other physiological traits (table S1). Furthermore, selected lines were again sown in the field in 2020–2021 and collect the seed samples to study the morphological and physiological traits.

### Determination of total starch content

For measuring the total starch content of 350 samples, Megazyme kit that was based on AOAC Method 996.11, AACC Method 76-13.01 was used. We followed the instruction mentioned in the kit for measuring the total starch content [36].

### Resistant and digestible starch

A commercial Megazyme kit (K-RSTAR 05/19, Megazyme Int., Wicklow, Ireland) was used for measuring the resistant starch and digestible starch from the flour of the wheat. Instruction was followed according to the kit guidance [36].

### Amylose/amylopectin

The amylose/amylopectin megazyme kit (K-AMYL 06/18, Megazyme Int., Wicklow, Ireland) was used for measuring the amylose content of wheat flour [4]. The protocol was followed according to the instruction of the manufacturer kit. Amylose and amylopectin were measured in percentage.

### Protein, gluten content and seed hardness

Grain analyzer had been used to measure the protein, gluten, and seed hardness. FOSS InfratecTM 1241 Grain Analyzer (FOSS Analytical AB, Sweden) was used for scanning NIR spectra. WinISI II v1.50 (InfraSoft International LLC, 2000) software was used to find out the final reading [37]. All these traits were measured in the percentage.

### Investigation of agronomic traits

Samples of seed from each mutant lines and WT were collected with three replications for measuring the grain width, grain length, grain area and thousand grain weight (TGW). SmartGrain was used for evaluating these traits by following methodology as described in previous studies [38, 39]. TGW was measured in gram, while width and length were measured in millimeters.

### Purification of starch for studying grain morphology

Seeds of mutant samples were crushed for extracting pure starch. The methodology was followed for starch purification as describe in somewhere else [11, 40]. Granule morphology was observed using a Nova NanoSEM 450 (FEI) scanning electron 133 microscope (SEM).

### Mutant lines exon sequencing

Selected mutants were used to capture the genetic diversity. The 4 mutant pools were subjected to exon capture and sequencing to compare the polymorphisms between different pools. The process of exon sequencing was followed as describe in Irshad et al.,[41].

### Expression analysis of mutant lines

Nineteen genes had been selected for quantitative expression analysis during different grain filling stages. These genes consist of starch synthase, starch branching enzymes, debranching enzymes, amylase, phosphorylase, and transcription factors. These genes play important role in amylose and amylopectin biosynthesis as well as total starch biosynthesis. Thirteen was main starch biosynthesis genes in which one was small subunit of ADP-glucose pyrophosphorylases (*AGPaseS*), while four starch synthase isoforms (*SSI, II, III, IV*), three were starch branching isoforms (SBEI, II, III) and remaining consisted of granule bound starch synthase (*GBSSI*) with three starch debranching isoforms and pullunase (*ISA1, ISA2, ISA3* and *PUL*). Further we selected four starch degrading genes (*Pho1, Pho2, BMY* and *AMY)*. Two transcription factors were also added for expression analysis that play role in starch biosynthesis (*SPA* and *TaRSR1*).

Expression analysis had been done in two low starch mutant lines (JE0089 and JE0418), two high starch mutant lines (JE0173 and JE0218), and the parent wheat variety ‘J411’. Samples of these mutants and parent variety were collected after 6, 12, 18, and 24 days anthesis (DAA). These samples were quickly put in the liquid nitrogen after taking from spike and then shifted to the − 80c refrigerator for RNA extraction. The detailed protocol of RNA extraction, cDNA formation and qRT-PCR followed by as described by Zhang et al., (2019). In addition, Actin (GenBank accession no: AAW78915), a housekeeping gene, was used as an internal control. Similarly, genes and their primer information was obtained from Singh et al., [14]. Three biologicals with two technical replications had been used for quantitative expression of these nineteen genes by using 7500 Fast Real-Time PCR System (Applied Biosystems, Forster City, CA, USA). The ∆∆CT method was used to analyze the relative expression level.

### Statistical analysis

Microsoft excel formulas had been used for calculating the mean and standard deviation. One-way analysis of variance (ANOVA) was used to study variation in total starch, in the replications for the mutant lines. To test the significant difference between mutants and parent variety for the above traits, Dunnett’s test had been performed. For the purpose of these analyses, three biological replications were used for studying all of these traits.

## Conclusion

In this study, 350 mutant lines were used and mutants with starch content variations were identified, this useful germplasm can be used for a genome-wide study and help to improve the starch-based nutritional quality in wheat. The gene expression of nineteen starch metabolic genes in two high starch mutants and two low starch mutants indicate that with starch main genes, there may be other genes (phosphorylases, pullulanases and isoamylases) responsible in starch biosynthesis. Further exon sequencing of these diverse mutant lines gives the information for further studies. There is a need to find out about starch metabolic genes through the backcrossing of these mutant lines and a developed NILs population for mapping.

## Electronic supplementary material

Below is the link to the electronic supplementary material.

Supplementary Material 1

Supplementary Material 2

## Data Availability

All data generated or analysed during this study are included in this published article [and its supplementary information files].

## Declarations

### Section

Study protocol complied with relevant institutional, national, and international guidelines and legislation.
