# Supplementary material for: Evaluation of altered starch mutants and identification of candidate genes responsible for starch variation in wheat
Source: BMC Plant Biol. 2023 Aug 1;23:377. doi: 10.1186/s12870-023-04389-3 (PMC10391901; doi:10.1186/s12870-023-04389-3)

**Figure S1:** Identification of mutations through sequencing. Allelic variations in stable mutant lines (JE0089, JE0418, JE0173, JE0218) (**A**) number of SNPs and Indels in the four mutants (**B**) number of transition and transversions mutation. (**C)** UTR_3, UTR_5, Frameshift mutations, non-synonymous and synonymous mutations. (**D**) Number of mutations in four mutant lines in A, B, and D genome.


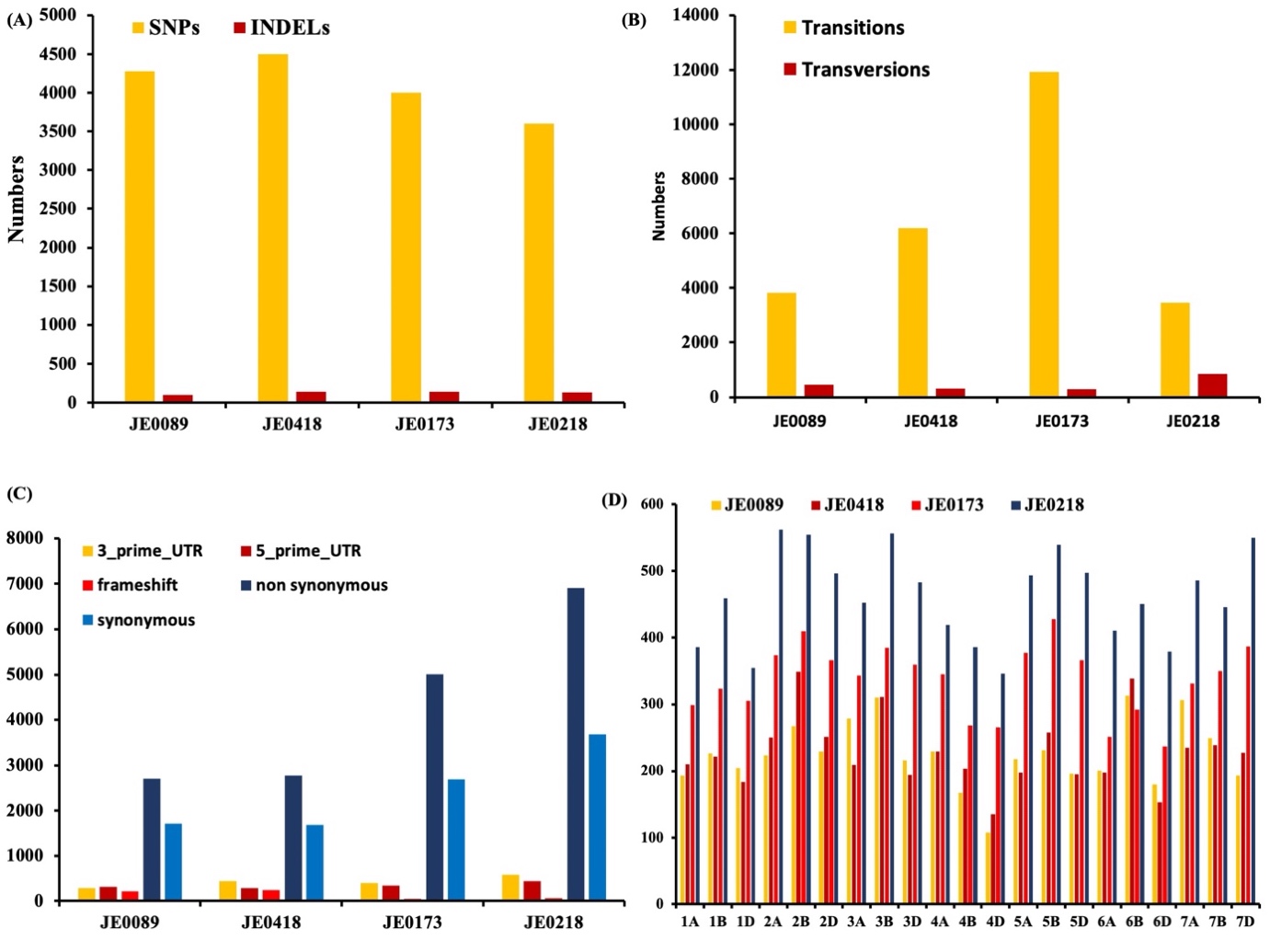

Supplement: Supplementary file 2 — Supplementary Material 2 [file 12870_2023_4389_MOESM2_ESM.docx]
